# Influence of lactose intolerance on colorectal cancer incidence in the Polish population

**DOI:** 10.1186/1897-4287-13-S1-A7

**Published:** 2015-09-09

**Authors:** Plawski Andrzej, Machtel Piotr, Pawel Borun, Marzena Skrzypczak-Zielinska, Arleta Wojciechowska-Lacka, Dariusz Godlewski, Tomasz Banasiewicz

**Affiliations:** 1 Institute of Human Genetics, Poznan, Poland; 2 Adam Mickiewicz University Poznan, Poland; 3 International Oncotherapy Center, Koszalin, Poland; 4 Center of Cancer Prevention and Epidemiology, Poznan, Poland

## 

During the last few years, a lot of efforts have been devoted to determine potential risk factors of colorectal cancer (CRC). One such agent which might increase susceptibility to sporadic CRC is an ailment of digestive system called lactose intolerance, since it negatively effects functioning of the intestines (after lactose consumption). It is caused by acidification of the lumen, osmotic balance disturbance, and an alteration in intestinal bacteria composition. Primary lactose intolerance is a genetic disorder caused by several loci, from which the most important for the Caucasian population is *LCT*-13910T>C (C/C - lactose intolerant phenotype, C/T and T/T - lactose persistent). It is located in intron 13 of the *MCM6* gene and operates as an enhancer of the *LCT* gene. The major aim of the following studies was to check a correlation between incidence of lactose intolerance and increased risk of sporadic CRC development. The studies rest on genotyping of *LCT-13910* loci in a group of control and 279 cases of sporadic CRC and comparison of frequencies of particular genotypes between those groups. Genotyping was performed by means of high resolution melting (HRM) analysis as a credible and fast genotyping method. Next, the results were subjected to statistical analysis by χ;^2^test of independence. The test, concerning association between lactose intolerance and sporadic CRC, achieved statistical significance. This observation may indicate the role of lactose intolerance as a risk factor for CRC (about 8% higher frequency of *LCT* -13910C/C genotype among CRC patients).

